# Book Reviews

**Published:** 1810-02

**Authors:** 


### CRITICAL ANALYSIS

### OF THE

### RECENT PUBLICATIONS

### ON THE

### DIFFERENT BRANCHES OF PHYSIC, SURGERY, AND MEDICAL PHILOSOPHY.

---

*An Essay on the Use of a regulated Temperature in Winter-Cough and Consumptions; including a Comparison of the different Methods of producing such a Temperature in the Chambers of Invalids.* By THOMAS BUXTON, M. D. Physician to the London Hospital and to the Surry Dispensary.

IT is now so generally understood that many of our English complaints arise from climate, that we cannot wonder if the means of obviating its inconvenience has very much engrossed the attention of the medical public. Indeed, there is one circumstance which renders the inquiry peculiarly necessary at this time. Such is the state of the continent, that an English invalid can find safety in no part. The island of Madeira seems now the only spot on which the panting phthisic can feel the balmy influence of an atmosphere suitable to the tender state of the lungs. The difficulty of finding accommodations in that highly favoured spot has long been complained of, and the increased resort thither must greatly out-run the accommodations which the inhabitants are now encouraged to prepare for strangers. Besides this, it neither suits the circumstances nor connections of many, to leave their friends and country. We cannot therefore fail to wish encouragement to every every attempt that may relieve these interesting sufferers without the necessity of encountering so many inconveniences and dangers.

On this account we shall follow our author according to the arrangement he has pursued in this useful little performance.

In his description of the winter-cough, we cannot help thinking Dr. B. a little too gloomy, as it is well known there are subjects who have continued through a long life with this inconvenience, and some of them have even apprehended the loss of their cough as the fore-runner of much more serious diseases. However, it must be admitted that such opinions ought not to be encouraged. A cough is certainly *morbus minime contemnendus*, and the neglect of it has been too often followed with all the inconveniences described by our author. The following remarks on inflammation of the lungs occurring in the spring, is sufficiently judicious and pointed.

" Inflammation of the lungs not unfrequently attacks a patient after he has suffered from winter-cough during a longer or shorter period; but generally after the lungs have been much weakened by a cough of considerable duration, and thus predisposed to inflammation. The patient catches cold, most frequently at the close of winter, or the commencement of spring. He feels, in consequence, acute pain in his side, or at the pit of the stomach. He perhaps spits up some blood occasionally, but usually in no large quantity; his difficulty of breathing is excessively great; his countenance is often livid. Sometimes he cannot lie down in his bed, or can lie only on one side. The quantity of phlegm which he spits up is very considerable. In a short time great debility comes on; the cough and the phlegm increase so much that the poor sufferer has no longer strength to struggle with the disease, and dies suffocated. The pain is often not very severe and acute, but rather consists in a sensation of a dull, heavy, oppressive nature, and spread over a considerable space. In this case likewise the cough and phlegm brought up are very considerable, and the debility, quickly induced, extreme, so that the patient's life is very soon destroyed. In the former case it appears that the membrane lining the exterior part of the lungs is the principal seat of complaint; in the latter the membrane lining the ramifications of the air tube. Yet from repeated observations I think I may venture to affirm, that neither of these membranes is in general the seat of severe active inflammation, without causing the other to partake in a greater or less degree of the same. The number of elderly persons who die in these ways is immense. I believe I have never passed a single spring since I began practice, without losing several patients from active inflammation attacking the lungs after winter-cough had some time existed. The patient, in these cases, generally, though not always, dies in a short time after the attack has commenced. These kinds of inflammation appear to me more distressing, both to the patient and to the practitioner, than any other, as the pain, or the oppression, of the patient are most most excessively great, the mode of treatment difficult to be decided on, and the success generally (to speak in the most favourable manner) extremely doubtful."

Dr. B. next proceeds to describe general dropsy, and water in the chest, as among the sequels of these complaints, after which he reverts to the causes of the original diseases and their remedies, considering a regulated equable temperature as the principal, and indeed as that without which the others will be altogether inefficient. This is illustrated with a variety of arguments, calculated, as the writer proposes, rather for the public at large than for the readers of our Journal. Several cases follow, related by different practitioners, and by the author, with some further testimonies.

The next consideration is the mode of regulating fire places in such a manner as to produce this effect with the least expence, both in the first fitting up, and in the future œconomizing of heat and fuel. With this view an accurate description is given of the German and Russian mode of heating rooms by stoves; after which the author describes his own plan, which, whilst it equally preserves most of the heat raised by the fuel, admits also some change of atmosphere in the apartment.

" Perhaps, says he, the best mode of showing the practicability of what I propose, will be by giving a description of Miss H's chamber, and of the stove by which it was warmed; as thus the mutual proportions of the one to the other will be immediately perceived. This room is thirteen and a half feet long, twelve feet wide, and eight and a half feet high. A common ironing stove was procured, twelve inches long, nine inches wide, and nine inches high. This, by my direction, was placed as far as it conveniently could be from the walls of the room, in order that every part of the room might be the more equably warmed. It accordingly stood two feet from the chimney-piece, which projected ten inches from the nearest wall. Its distance from the next nearest wall was five feet. A chimney-board was made by a carpenter to fit into the fire-place vertically. In the upper part of this a hole was cut, through which the flue of the stove passed. The stove was fixed so low, that the flue, which had an elbow almost immediately after quitting the stove vertically, then passed nearly, but not quite, horizontally, as it gradually ascended from the stove to the hole in the chimney-board. The stove stood in a flat iron dish, with the rim very slightly raised. This was placed on the floor, and projected for some distance round the stove, to hinder the cinders which occasionally fell out, from setting fire to the boards. I have entered into these particulars, that they may be a guide to any person who wishes to put up such a stove in a chamber. At first some little inconveniencies were found in the management of the fire. But in a day or two these were completely overcome, so that, although the weather during the spring of this year (1809) was very severe, the chamber could always be kept with the greatest ease at a temperature from 60° to 65°. In order that that this temperature might be strictly preserved, a thermometer was always hung up close to the head of Miss H's bed. Notwithstanding the heat was thus kept up, a person entering the room from the open air, could not perceive any unpleasant, close or confined sensation. It is worth while noticing that the fire never smoked, a circumstance of some consequence in a sick room, particularly where the lungs are affected.

"From the large heated surface of the stove, constantly surrounded by the air of the chamber, as well as from the distance which the smoke has to travel before it ascends the chimney, a much smaller quantity of heat uselessly escapes up the chimney than in the common English fire-places. Hence we may easily credit, that the part of the room farthest from the stove could be kept at an equable temperature of 60 or 65 deg. The air of the room was constantly changed, although not with the same degree of quickness with which it is changed by the common fire-place; for the opening into this stove is only of sufficient width to let the air enter which is necessary for the fire. A sufficient change thus takes place to keep the air pure in a moderate sized room, without occasioning those strong currents and drafts, so troublesome in our wide open fire-places. But if from accident the air at any time should appear at all vitiated, the draft may easily be increased, and the room ventilated by augmenting the fire in the stove, and opening sufficiently the door or window. The expense attending this kind of stove I have stated at three pounds ten shillings, which cannot be considered as a very extraordinary sum; and the labour of putting it up is inconsiderable. The trouble of preserving the heat of the stove is not greater than that of keeping up a common fire; and the expence is not so great, as it does not consume so much fuel. The principal trouble, attendant on a regulated temperature, is, that some person must look after the fire by night as well as by day. But this trouble is incident not merely to the mode which I am now recommending, but to every mode of preserving the temperature constantly at an equal standard. The plan adopted by the friends of Miss H. was, that some one of the family sat up late, and just before going to bed raised the fire, which occasioned no danger, as the extensive iron plate placed around the stove on the floor, effectually secured the boards from accidents. Another of the family rose early in the morning, and immediately kindled the fire in Miss H's stove. By this management the thermometer rarely sunk considerably during the night, being seldom in the morning much below 60 degrees. But still it must be confessed, that, if practicable, it would be better were the fire kept in during the whole night, as thus the thermometer need never descend lower than the standard; for every degree below that is a deviation, greater or less, from the plan proposed. The expence attending the first establishment of this fire-place I consider as the only additional one, and the trouble of a person sitting up as the only additional trouble. It is true, that in the method adopted by Miss H's friends, the plan was not carried to its greatest perfection; but this near approximation was infinitely better than a total neglect of the plan."

The only objection we have to this plan is, that the materials of the stove not being specified, are we conceive of iron, which always produces an unpleasant smell. There is one made of four pieces of porcelaine, placed in such a manner as to form a hollow square, on the top of which is placed another flat piece, as a cover, the whole is bound together by brass hoops. Such a one as this is well known as forming part of the furniture of Sir Joseph Banks's parlour, in which he receives his morning calls, and is so placed that the frequent opening of the door is scarcely perceived. We ought to remark that Dr. B's great attention to economy in the construction of his stove, is probably the only reason why he has not adverted to such materials, as he does not fail to give the preference to the composition of the Russian over the German stoves, because the former are of iron. But we conceive the different expence could not be considerable, where the object, being only health and convenience, the coarseness of the earthen ware would not be an objection.

---

### Edinburgh Journal. No. XXI.

Article 1.—*Medical Report for Nottingham, from March* 1808 *to March* 1809. By James Clarke, M. D. Physician to the General Hospital, and to the Vaccine Institution.

The first part of this paper consists of the epidemic constitution of the air for that period. Valuable as these registers always are, the present contains nothing particularly interesting to the general reader. A case of hydrophobia follows, concluding in the usual melancholy way. The only thing worth remarking is, that though two or three other people were bitten, yet in the course of twelve months, no threatening symptoms had appeared on either of them. In the deceased, no inflammation or uneasiness on the wounded part was ever felt subsequent to the injury. The dog showed no rabid symptoms. Though this seems to excite much surprise and even doubts in some writers, yet it has been very often observed before.

Article 2.—*Observations on Purulent Ophthalmia.* By William Goodlad, Member of the Royal College of Surgeons in London, &c.

Article 8.—*Observations on the Cause of Purulent Ophthalmia of Infants.* By W. Ankers, Esq. London.

Article 9.—*On the Purulent Ophthalmia of New-born Infants.* By Robert Lyall, H. Surgeon.

As this subject has lately been somewhat minutely discussed in both the Journals, we shall offer only a brief statement of the contents of these papers.—Mr. Goodlad goes chiefly to remind us of what has been first remarked as an inference from Mr. Hunter's account of of the transplanted teeth, namely, that the healthy secretions of one animal, applied to the secreting surfaces of another, even of the same class, may, in certain cases, become morbid poisons. The paper concludes with proposing a division of the inflamed vessel, as the best remedy. This gentleman also uses fomentation of poppy water; and to the palpebræ, when they continue thickened, applies any stimulating ointment. Mr. Ankers follows pretty nearly the same ground, as to the cause of the disease; and remarks, that having always carefully attended to an early ablution of infants' eyes, immediately after birth, he has never met with a case of purulent ophthalmia in the offspring of females delivered by himself.

Mr. Lyall gives an abstract of all that has appeared lately in Gibson, Ware, Scarpa, and the two Journals. He seems disposed to agree with all, excepting Mr. Simmons, who, by his late communications, does not appear to us at all obstinately tenacious of his opinions. Lastly, Mr. Lyall having admitted all the causes proposed by the different writers, concludes by recommending the remedies of each.

ARTICLE 3.—*Observations on the Fever which appeared in the Army from Spain on their Return to this Country in January, 1809.* By JAMES M'GRIGOR, M. D. Inspector of Army Hospitals for the Portsmouth, Severn, and South West Districts.

FEW papers are so important as reports of diseases and remedies on that scale, which army practice affords; yet of how few are we in possession which add to our stock of real knowledge! Every older practitioner seems, with a few exceptions, as if relating a muster roll, and the juniors are so full of the success of their remedies, as to deem it unnecessary to *describe* accurately the diseases they *cured.* The paper before us being drawn up by an Inspector of Army Hospitals, might be expected to be free from both these imperfections, and to a certain degree it is less chargeable with them. But Dr. M'Grigor, though we believe an honest man, a well-informed and experienced practitioner, and even in the habit of writing, has not yet acquired the knack of laying statements before his readers, in such a manner, as to preserve a proper interest, by connecting the various facts, nor of course to teach him to derive that practical knowledge which is the main object of all medical reading.

The "Observations" include the practice of not less than a dozen medical gentlemen of different departments; yet so confusedly are they related, and most of them with such brevity, that it will be difficult to form any decision, why some succeeded with remedies altogether different, and others failed, though apparently with a practice similar to their more successful brethren. From the materials, such as they are, we shall endeavour to draw every advantage that the manner in which they are thrown together admits; trusting to the candour of the author and of our readers, if we sometimes seem to infer more than can with certainty be collected

 from from accounts, which, in our opinion, the writer has taken too much pains to compress.

Dr. M'Grigor begins by stating the health of the troops in Portsmouth district, before the arrival of the wreck of the Spanish army, and the unfavourable circumstances under which the latter were received.

"The healthy state of the troops, during the winter quarter, as shown by Table No. I.* in the districts where I have the medical superintendence, was interrupted by the arrival of the wreck of our unfortunate army from Spain. The first transports with them arrived at Portsmouth, about the 20th January, when they appeared more the victims of disease than of the sword of the enemy, severely as they had suffereed by that.

"The unfortunate circumstances under which this army was placed, will readily account for the appearance of a mass of disease, and of the worst character. A dispirited retreating army, in which order and discipline, so necessary to its health, were with difficulty maintained, badly clothed, marching over a desolated country, in the most inclement season of the year, was readily predisposed to disease. In their retreat, the British army mixed with, or followed in their route, the remains of that army which the Marquis de Romana had transported from the shores of the Baltic; a fever of the most malignant character had committed great havoc in Romana's army, and the contagion of it was readily communicated, under the above circumstances, to the retreating British army under Sir John Moore. It has been said, that this army had likewise been debilitated by marches, harassing and long, beyond their physical powers; and it is acknowledged on all hands, that they suffered much by irregularities on their march, and an indulgence in intoxication the most disgraceful.

"After the action of Corunna, the army, imbued with disease, some corps more, some less sickly, was thrown on board transports, and mixed together. During the voyage to England, which proved a very tempestuous one, it was not easy to keep up ventilation, and to preserve that state of cleanliness of the persons of the men, as well as of the ships, which is at all times so necessary to the preservation of health afloat.

"Under such an accumulation of misfortunes, it will not appear surprising, that, as it arrived at Portsmouth and Plymouth, every part of the army was found to be unhealthy. It was observed, however, that that part of it which, under General Crawford, embarked at Vigo, was much more healthy than the main body which embarked at Corunna; indeed, the light corps who embarked at Vigo,

* By this table, it appears that the whole number on the sick list, including venereals and slight complaints, was 1644, the strength being 12,284, and the number of deaths from November 6, 1808, to February 11, 1809, was only 25. Ed.

Vigo, continued tolerably healthy, until, at Portsmouth and other quarters, they mixed with the other divisions of the army.

"It was not, however, only in Spain, or on leaving its shores, so fatal to Englishmen, that Sir John Moore's army was unprosperous. Misfortune continued to pursue them to their own shores. So tempestuous was the weather for some days after the ships had arrived at Portsmouth and Plymouth, that it put a stop to all disembarkation and communication with the shore, while disease was hourly making rapid progress in every ship of a very large fleet.

"But as it is probable that the whole of the misfortunes of this brave but ill-fated army were induced by causes which no human ability could have foreseen or averted, let us not, at this period, attempt to withdraw the veil with which time is fast covering them.

"Would that we could so far profit by them, as in future to prevent their recurrence; or, if such misfortunes are inevitable in the present state of human affairs, at least to mitigate the miseries of the soldier! What could be done by the gentlemen of the medical department in Spain, under circumstances the most arduous, I firmly believe was done; I willingly bear public testimony to the great zeal and humanity of these gentlemen on landing at Portsmouth, as well as of the other medical gentlemen employed to attend a sick army here. Theirs was a service of as real danger as any that had occurred to military officers in Spain; and it is much to be lamented, that so many of them fell a sacrifice to the zealous and unwearied discharge of their duty here."

We shall now endeavour to select such passages as will best mark the character of the fever, particularly as far as it was seen by Dr. M'Grigor himself.

"On the arrival of the first ships, several died in the boats which brought them on shore, and were brought corpses into the hospitals, while the greater part was in the last stage of low fever. It is my duty likewise to mention, that, for some time after their arrival, the relapses, both in dysentery and fever, were extremely frequent; and that, though the conversion of these diseases frequently occurred, yet, as this could not be in every instance ascertained with accuracy, it is not noticed at all in the table. It will be evident, that, if these circumstances were introduced, they would greatly lessen the proportional mortality.

"In the symptoms, the fever which was prevalent, varied much at the different periods at which it appeared, and in the different description of subjects it attacked. At first, in some cases which I saw on ship-board, and in the first cases which were received into the hospital, most of them being in an advanced stage, had the symptoms of what is described a nervous fever, with not a few of the appearances said to denote a state of putrescency, the body being covered with petechiæ, maculæ and vibices, and there appearing (as was reported me) in a few cases, glandular or bubonic swellings. This was the case with those first received into the naval hospitals, into the two hospital ships, into the depôt hospital at

 Hilsea

Hilsea, and at the general hospital at Gosport, particularly with about one hundred cases received there from Hilsea, when this station overflowed.

"In the cases of several officers first landed (I understood that the same had frequently been observed in Spain), the pulse was found but little altered from the natural state, and an inattentive observer would think the patient ailed little; but while there was not great prostration of strength, and even when food was called for, an obscure low delirium might be discovered, with a tendency to despondency and melancholy.

"A prominent feature of the first cases of this fever, which made their appearance on ship-board, or immediately after landing, as well as in those who had been attacked in Spain, was the strong disposition to gangrene in the feet and legs. This was, most probably, induced by the previous circumstances of great fatigue and long marches, undertaken by the exhausted soldier, where, in many instances, he was badly provided with shoes. The tendency to mortification in the sacrum and back was likewise great, and phlegmonous abscesses frequently appeared on other parts of the body. In some cases, erysipelas was seen; but the most constant symptoms in this fever was the great determination to the head and chest, with, in many cases, a torpor of the abdominal viscera.

"The cases which made their appearance while the troops were in harbour here, or immediately after landing, had, I believe, almost universally topical determination, in a greater or less degree; the head was very frequently affected, but more frequently the lungs and pleura. In the progress of the disease, imperfect hearing and bluntness of all the senses were frequently attendant symptoms.

"It was not a little curious to observe the different appearances which, at different periods, in the various subjects of its attack the disease wore. As already mentioned, the cases which were first landed, and they were mostly in an advanced stage of the disease, all appeared to be pure typhus. Those which made their appearance after the ships came to anchor here, whose first stage we had an opportunity of seeing, had a considerable degree of re-action, with generally topical determination.

"The orderlies or attendants on the sick were provided from the 8th Royal Veteran Battalion, or from the regiments of militia in garrison here. A considerable number of these orderlies were attacked with the fever; and in all these corps several cases of it appeared, but with varied features. While the old invalids of the 8th Royal Veteran Battalion, who suffered much by this fever, had it with its lowest symptoms, yet, in almost all of these men, catarrhal or pulmonic symptoms were seen. In the healthy and robust frame of the militiamen, as well as in the men of the German Artillery, the disease, when it appeared, assumed a very different character. They, in the first stage, had always the strongest arterial action, with great determination either to the head or to the chest; chest; in many cases bordering closely on pneumonia, where the great and only relief was by venesection and evacuants, the blood, after the second or third operation, always shewing what is called the inflammatory crust."

Such, as far as we can make out, is the result of the author's own observation, or information immediately derived from others. It is not less a matter of surprize than regret to us, that we can no where discover what was the author's practice. It is probable that as he was inspector, the practical part was left to others, but still it was, if not under his direction, at least under his controul, which might have given the best comparative means of forming a true judgment of the result of different methods.

We shall now extract, with as much accuracy as we can, the practice of the other medical gentlemen, in which Dr. Macgrigor is somewhat more communicative.

Dr. Keils, of the King's German Artillery, observed inflammatory action in most of the cases which occurred in the hospital, of that corps. He used the cold affusion, and no fatal case occurred.

" Mr. Fosbrooke, surgeon of the Durham Militia, reports, " that all the cases which he admitted into the Durham Hospital were the synochus, and that some of them closely approached to synocha. They commenced with strong arterial action, the patient having the appearance of inflammation going on in one quarter or other; but after a few days, there appeared a tendency to typhus. When petechiæ were seen, the excretions were scanty and fetid, particularly the alvine; in most cases dark, in some seemingly mixed with blood."

This gentleman reports, that he tried the cold affusion in the Durham Hospital, " that it succeeded in no case; nor was Dr. Hamilton's plan of treatment with purgatives more successful in the hands of this gentleman. Mr. Fosbrooke, with success, gave his patients a combination of calomel with antimonial powder, in repeated doses. It will be recollected, that all the cases in the Durham Hospital had a mixture of pneumonia; and I may here mention, that my friend, Dr. Alley of Cork, has met with the greatest success by using mercurial frictions, in the disease which some have denominated pneumonia typhodes. Nearly two years ago, when this disease and an epidemic catarrh were very prevalent among the troops, I witnessed the great success of some gentlemen who used calomel, or calomel and antimony, so as to affect the gums. I was a few years back led to this practice, by a paper sent me by my venerable and much respected friend, Dr. Wright of Edinburgh."

Mr. Arminger in his report, says, " In the first attack, with extreme langour and lassitude, there were severe aching pains in the head, back, and large joints. As the disease advanced, the temperature during the exacerbation rose very high, (not determined by degrees for want of a thermometer), especially in the cases which did not come under my treatment till the third or fourth day of the disease. The heat was pungent, the sensation to the patient, as well as to the observer, burning, in most of the cases pretty equally diffused; but in those in which the determination to the head was greatest, the lower extremities, particularly the feet, were generally deficient in warmth, sometimes very cold. The pulse frequent, and certainly both harder and stronger than in the typhus mitior of Cullen; the urine deficient of high colour, and remaining long unaltered in its appearance; considerable determination to the head, as evinced by fulness and flushing of the countenance; head-ach of the throbbing kind; vessels of the tunica adnata turgid; temporal arteries seem to pulsate strongly; delirium of the more lively kind; increased susceptibility of impression; correspondent impatience and restlessness; want of sleep. When the determination to the head was still greater, it was characterized by drowsiness, stupor, delirium mite; and, in cases which ended fatally, coma, floccitatio, and subsultus tendinum.

Mr. Arminger concludes a very accurate report, by observing, "that during the period of the prevalence of this fever, agues were more frequent;" and he adds, "the majority of the cases of fever which I saw were the synochus of Cullen; but I feel disposed to class some with remittents."

"Mr. Arminger observed, that when mercury was employed, the febrile action seemed to yield as the mercurial action was established."

"This gentleman used purging on the admission of his patients, both to remove the torpor of the abdominal viscera, and to relieve the determination to the head.' Mr. Arminger sometimes used the aspersion with cold water, and sometimes cold affusion. He says, 'that no case proved fatal in which mercury had a fair trial. It seemed to produce great good when the determination to the head induced stupor. In two cases in which the usual means had been carried to their full extent previously to its use, and little or no benefit had been derived, he considered it as the curative means; in several other cases, it was usefully employed as an auxiliary."

"Dr. Clarke, who had latterly the sole charge of the General Hospital at Gosport, and who at first had the charge of the majority of cases of fever there, writes me, 'that, in the eighty cases under my charge, which came from Hilsea, it appeared pure typhus gravior; but, from what I have since seen, I suspect, that even those cases had, in the beginning, every symptom of synochus. They were of from eight to ten days standing; the symptoms of debility and putrefaction were strongly marked; there were petechiæ or vibices in almost every case."

"Dr. Clarke says, that on their admission into the General Hospital, every patient was put into a warm bath, and made thoroughly clean; they were then freely evacuated by purgative medicines, and frequently the fœtor of their stools was intolerable. Whenever the heat of the surface was above the natural standard, standard, the cold affusion was freely used; this degree of heat was often induced artificially by stimuli given internally. I recollect one case in particular, where, on admission, the pulse was hardly perceptible; the extremities were cold, and the patient appeared to be rapidly sinking. After the warm bath, he was put to bed, and, as deglutition was easily performed, he had stimulants freely given; the pulse then rose; the heat of the surface became intense. In this state, the cold affusion was instantly used, and with the happiest effect; the disease ran its course, but the symptoms were comparatively mild. Wine and other stimulants were given in every instance, so as to keep up sufficient action. This required much attention, as the danger of giving too much appeared in many cases more alarming than the disease.

"There were three very interesting cases among the eighty which I received from Hilsea, which, on their admission, had hardly any marks of vital action; the face, hands, and feet, were livid; the strongest stimulants were in those cases applied, such as general friction with volatile liniment, sinapisms, &c.; internal stimulants, when deglutition was practicable, were also used. Two of these three recovered; but in one of them the toes sphacelated. Of the whole eighty cases, I think four or five died during the twelve days I had charge of them; the others seemed in a state of convalescence; but I believe a few relapses took place.

"The appearances and treatment were much the same on board the hospital-ships; there were but few cases of fever, and I do not recollect one fatal case. In none of these instances was the lancet employed; but I have since used pretty freely, and with success, whenever there is any symptom of local affection, such as pain in the head, thorax, or abdomen, even where the pains in the extremities are much complained of, and the arterial action considerable. In those cases, the typhoid type has seldom appeared, and I am well satisfied of the utility of the lancet in pyrexia, where there is the slightest symptom of local affection. You are not unacquainted with my own case, in which the lancet was so freely used; and I yet reflect on the relief I felt at each bleeding, with gratitude to the able advisers, Dr. Cabbell and Mr. Burnett. I had only once an opportunity of using the cobweb since I learned from Dr. Jackson the extraordinary properties of that substance; it was in the latter stage of chronic dysentery, where there was much anxiety and great irritability; and in that case, it certainly had all the good effects attributed to it by the doctor. The patient became quiet and easy immediately after taking five grains of it."

"Dr. Keating, who was in charge of the Depôt Hospital at Hilsea, to which, as well as to the Naval Hospital, most of the severest cases which were first landed were sent, reports of about two hundred cases which he had, that almost every one of whom came to him covered with petechiæ." This gentleman used cold affusions with cordials, as recommended by Dr. Jackson.

Mr.

Mr. Foaher of the West Essex, describes the disease as varied according to the subject, and the period at which he saw it. His practice seems to have varied accordingly. The following is all the information we have of the dissection.

"There was some variation in the appearances found on dissection of the dead. I regret that the regulations of the Naval Hospital did not allow our dissecting but a very few of the cases which died there. In all the other hospitals, agreeably to the rules which I established ever since I have had any charge, a dissection was made of every case which died, and, with a very few exceptions, this was done.

"The reports of the dissections made at the General Hospital, under Dr. Clarke and Mr. Aveling, describe in several cases an affection of the brain being observed, the vessels in each hemisphere being found turgid; water frequently in the ventricles; the substance of brain in some case soft and pulpy. On opening the chest, both lobes of the lungs frequently bore marks of previous inflammation; in some cases, adhesions of the pleura, with some water in the pericardium.

"The tenor of the reports made from most of the hospitals was the same; but in those which I have from the 8th Veteran Battalion, West Middlesex, and Worcester Militia, no morbid appearances were seen either in the head or chest."

We shall intermix our remarks with the remainder of our extracts, to render the winding up of the whole as pointed and as useful as we can.

And first, we are extremely glad to find that somebody besides ourselves, is disposed to quarrel with a part of the medical language, which, in the opinion of one of our brother labourers, has been attended with such serious inconveniences.* If it is necessary to give diseases names, they should at least be such as mark one striking character, and we should be careful, that even that name shall not mislead us, by teaching us to look for such a character as in all cases so uniform, and attended with symptoms so correspondent, as in all to require a similar mode of treatment.

It may be said, that fever is a general term, yet we admit of different kinds of fevers, and a different treatment to each.—Time was, when we were satisfied with the term fever, and the immortal Sydenham thought it enough to describe each as it appeared, and to direct the mode of treatment accordingly. But since we have heard of *typhus* as a contagious disease, arising from camps, poor houses, and prison, and also of typhus as the term for the low nervous fever, arising from any cause; what can we expect, but that every low fever will be called typhus, and every typhus a low fever? Hence, should the infectious atmosphere of ships, camps,

* Adams, on "Morbid Poisons," p. 375 and 376.

camps, or prisons, induce a high fever, we are obliged to halt, in order to determine whether we shall call it typhus, or whether the atmosphere, supposed to be peculiar to typhus, may not induce synochus or synocha, or some other name imposed upon us, whilst we ought to be considering every symptom, tracing every probable cause, and accurately attending to the effect of remedies.

If typhus were to be the general name for every fever, arising from an atmosphere vitiated by confinement of the sick, and for that only, there would be no more impropriety in the use of it, than in the use of variola for small-pox, a disease which arising from the same effluvia, shows different symptoms, and is treated according to those symptoms. But it is evident, that typhus not only implies a fever from vitiated atmosphere, but a fever of a low type. Hence, if such an atmosphere should induce a high fever, either the terms must be abandoned, or the practice must be in direct opposition to the language. Having said thus much, we shall extract a few passages from Dr. M'Grigor's paper in illustration to our meaning, and add some further remarks, which we trust may be useful to our younger readers intrusted with the lives of our brave defenders.

"Though, says our author, as first seen here, when the army disembarked from Spain, the fever had low nervous, and likewise putrid symptoms, and was called typhus, (a term in by far too general use), yet, in most of the cases, it was of a very different type; and several gentlemen were of opinion, that, though in the cases first landed here, the typhoid symptoms were seen, that the disease had commenced with those of a different form of fever. Neither in the case of Dr. Clarke, in that of Mr. Lind, surgeon of the 43d regiment, nor in that of Mr. Foaher, surgeon to the West Essex Militia, was the fever of the typhoid type, though they were attacked with the disease during their attendance on the sick from Spain. In all of them, there was strong arterial action, and great topical determination.

"I have heard, that, in the cases of some other of the medical gentleman attacked, the symptoms were those of nervous fever. I saw that this was the case with Mr. M'Grigor, surgeon of the Military Asylum, who volunteered to act as staff-surgeon here, in Mr. Aubert of the Guards, and in that of Mr. Hawkins of the Oxford Militia."

Of this passage, we shall only remark, that the gentlemen mentioned in the first paragraph, were exposed to the atmosphere of the sick, whilst they themselves were probably in high health, having suffered no previous privations, anxieties, or repeated causes of dejection. As to the gentlemen under the immediate inspection of Dr. M'Grigor, whose symptoms were those of nervous fever, we should have been glad to know whether, like the former, they were exposed to the sick on their first landing, or not till the atmosphere with which their persons and cloaths were imbued, was somewhat less concentrated by exposure to the air, and the the cleansing the bodies of the patients; or not till they had been previously reduced by a long attendance on their duties.

"I may in this place," says Dr. M'Grigor, "mention further, that I learn from my friend Mr. Burnett, who superintends the hospitals for prisoners of war in this quarter, that at the same time that our army landed from Spain, many prisoners were brought over, whose disease appeared to be the same fever as that under which our soldiers laboured. Mr. Burnett had frequent opportunities of seeing this disease on a very large scale. It was this gentleman who, in conjunction with Dr. Cabbel, had the treatment of Dr. Clarke, whose case, drawn up by himself, is subjoined. Mr. Burnett says, in a statement with which he has favoured me, "that he has no doubt of this disease having been inflammatory from the beginning. I accordingly treated it with liberal evacuations, and in no instance, when the patient came under my care in his first attack, did I fail of producing a complete remission in twenty four hours. Though the disease did, in some instances, assume the form of synochus, it was only in such patients as had not been evacuated in the early stage of the disease. *I had about fifteen or sixteen of my nurses taken ill; they were bled to sixty or seventy ounces the first day they complained, and none of them died.'* Mr. Burnett's authority is of considerable weight. The prisoners of war here are seldom under 10,000; and in the hospitals under Mr. Burnett there are seldom fewer than three hundred sick.

"I must not conceal, however, that the fever prevalent at this time appeared with features extremely different in other quarters. In an accurate memoir of Dr. Lemprere's, which he was so kind as to permit me to peruse, I see that the cases received into the Depôt Hospital, Isle of Wight, were pure unmixed typhus."

It is not easy to ascertain the exact situation and description of the other patients; but one thing we may see, that the nurses, who, like the orderlies in the last account, were exposed in a similar manner to the destructive cause, and affected in the same manner, were relieved by the same means.

"In the hospital for the prisoners of war here, [Woolwich,] Mr. Burnett says, "I have had patients sent to me on the 8th or 10th day of the disease, that had been taking bark and camphor, under the idea that the disease was typhus, with high delirium, foul tongue, strong full pulse, and passing their stools and urine involuntarily. I have ordered them a bleeding of twenty ounces, blistered the head, and given a cathartic; and on the following day have found my patient calm, in one case without pyrexia, and, by persevering in this plan, they ultimately recovered. Those who were not bled in the early stage of the disease, were frequently subject to cynanche parotidea, which was always a favourable, though a very painful symptom. I have found it indispensably necessary to attend to the state of the bowels during their convalescence, and their diets required equal attention, as constipation on the one hand, and a full meal of animal food on the other, has, even after five five or six days of convalescence, induced a return of pyrexia. You will readily recollect our worthy friend Clarke's case, and how fortunately it terminated: you know we were not sparing of the lancet, which I can have no doubt saved his life."

Here we find nothing but bleeding would relieve even prisoners of war. What circumstances gave rise to such high inflammation in such subjects, we cannot learn from the relation, but Dr. Clarke's situation is more easily accounted for; and we shall conclude with the history of his case as given by himself.

"Dr. Clarke's Case.

"First symptoms, lassitude, great prostration of strength, loss of appetite, vertigo, dimness of sight, head-ach very severe, with strong pulsation of the temporal artery, thirst, and every other symptom of pyrexia: bowels, formerly regular, now constipated to a degree, and remarkably torpid. After taking repeated strong laxative and cathartic medicines, without the least effect; at last, by a large dose of jalap and calomel, two or three evacuations were procured, with considerable relief. In a few hours, all complaints increased, and, at my own earnest request, I was bled in the arm; but before sixteen ounces were obtained, syncope was induced. I continued much in the same state; pain in the head, and want of sleep, with extreme anxiety, my chief complaint. Was bled to sixteen ounces this evening, independent of the bleeding on board of ship, and was kept in a constant state of nausea by vin. antim. in frequent doses; this invariably eased my head-ach, but vomiting was once occasioned by it, which exertion brought on such action, that the pain became excruciating, (but very slight, if any, intolerance of light); head-ach continuing very severe, notwithstanding the hair being removed, vinegar and water constantly applied, and a variety of internal medicines; a dose of camphor was taken one night, which brought on great delirium, and much increased pyrexia. At the suggestion and strong entreaty of my good friend and excellent practitioner, Mr. Burnett, I was freely blooded, with almost instantaneous relief. The bleeding was repeated as often as the head-ach returned, and each time with the same good effect. In all, I was nine times bled, of which three were on the 11th or 12th day from the first attack. I lost about 127 ounces of blood. With the exception of the delirium brought on by the camphor, I was quite collected during the whole period, except when I thought I felt disposed to sleep. I then had much watching, and frequent incoherent ideas, but very seldom gave way to them, I mean by making unconnected remarks to the attendants. No low or typhoid symptom appeared throughout the whole course of the disease. In the early stage of convalescence, I was allowed nothing stronger than milk, tea, weak chicken-broth, and the like; nor did I take any thing stronger during the whole length of the disease. For nineteen nights and days, I cannot say I was ever sensible of being asleep, nor could any of the attendants attendants ever find me so. Towards the latter end, or from the 12th to the 19th day, want of sleep was the only complaint; and, for several weeks after this period, I never slept above an hour or so at a time. Vertigo continued on the least exertion for some months.

(Signed) J. CLARKE. M. D.

*Portsmouth, 20th August*, 1809.

We wish our readers to attend to various accounts of cold affusions in the above paper, and to determine for themselves, whether mere heat is a sufficient indication for that remedy, or whether the preparatory steps recommended by Dr. Jackson are not sometimes necessary.

ART. 4.—*Lues Bovina Intertropica, and the Consequences thereof, with Remarks. By* C. CHISHOLM, M. D. F. R. S. &c. &c.

"In the year 1783, in the island of Grenada, in the West Indies, a very singular coincidence took place. Late in that year, the cynanche maligna appeared in several parts of the island, for the first time observed, I believe, by the oldest inhabitant in that or any other of the West India islands. The symptoms of this disease were most violent; its rapidity to a fatal termination most alarming. But the circumstance which gave greatest singularity to this disease, was its concomitancy with a contagious distemper, of a very extraordinary nature (within the tropics) epidemic among the cattle and mules in the same parts of the island, wherein the cynanche maligna appeared. Both were new and unknown, and both were concomitant; insomuch, as to render it difficult to perceive whether they proceeded from a cause common to both, or whether the cynanche was an effect, on the human race, of an imported contagion, which seemed peculiarly, in the first instance, to affect the horned cattle and mules. These animals, while feeding, and apparently in perfect health, in the pastures, suddenly fell down dead. The malignity of the disease had so rapid a progress, that seldom could other symptoms, or rather any symptoms be observed: sometimes, a few minutes before death, the animals were languid, lay down, and neglected their food. Sometimes a swelling of the glands of the throat formed a large tumor, which might be perceived for some days before death; but though this swelling sometimes suppurated, and though the matter was discharged, it never proved critical. On dissection, the whole course of the trachea or œsophagus, the stomach, and greater part of the intestines, were found in an inflamed or a gangrenous state. Various modes of cure were adopted, but, except in a few cases, always without effect. In these few excepted cases, the Peruvian bark, given in very large quantity, seemed to complete a cure; but the use of this medicine was too expensive to render it extensive, and the instances I have mentioned, I believe, were experimental. Methods of prevention were also tried; of these, I was assured, that tar rubbed on the forehead, to the nose, and under the throat, had frequently the desired effect. At this period, 1783, Grenada had intercourse only with some of the other islands islands in possession of the French, within the tropics, and with Ostend in Austrian Flanders chiefly, in Europe—there was no intercourse with North America, and none whatever with the Spanish colonies; and, upon the whole, well-grounded reasons exist for believing that the fomes of the bovine pestilence was imported from Ostend, by the Imperial neutral ships which exclusively carried on the only trade the circumstances of the existing war then permitted.

"On those plantations where care was taken to burn the carcases of the diseased cattle, no further consequences resulted. But these unhappily were few. On those where this precaution was not used, and, indeed, it is surprising that it should be used in any, seeing that the disease was new, and its effects unknown, the flesh of the cattle that died being dug up, and eat by the negroes, proved most dreadfully septic, producing a pestilential carbuncle, attended by a malignant fever. There were not wanting instances of the iniquitous practice of offering the flesh of diseased cattle for sale, and on these occasions such was the highly septic nature of this poison, that even touching the flesh, in such manner as that part of the sanies adhered to the finger, produced the same fatal consequence. A remarkable instance of this occurred in a respectable married lady of the island. In the finger to which the virus was thus inadvertently applied, a pestilential carbuncle appeared, and her life was preserved by the amputation of the diseased member.

"This disease, thus originating, was distinguished by the name of malignant carbuncle; and among the French part of the population by that of *Charbon*. The series of its symptoms was thus: Without any previous symptom of disease, the patient complained of a tumor, often in no certain part of the body, but generally on one cheek, resembling the inflammatory vesication which succeeds inoculation for the small-pox. Soon after a fever came on, but by no means violent, and continued during the twenty-four or thirty-six succeeding hours, when it gradually subsided, and left the patient apparently without a single symptom of disease except the tumor. This tumor was nearly circular, had a depression in the middle, and the skin immediately around it was œdematous. At this period, however, in the middle of the tumor, a small whitish carbunle arose, and breaking discharged considerable quantities of a yellowish ichor. But this seeming freedom from disease was, in about twenty-four hours after the eruption of the carbuncle, succeeded by vertigo, a most excruciating pain stretching across the abdomen, accompanied by anorexia, thirst, and palpitation of the heart; the pulse sunk below the natural state; cold sweats broke out; and in short, the patient was carried off in twelve hours after the seizure of these latter symptoms. It is obvious, that the danger of the disease lay chiefly in its obscurity and novelty—for among the negroes more especially, being much subject to sores, sometimes attended with slight symptomatic fever, ver, and which were easily cured, no apprehension of fatal consequences, for some time, was excited; and when the second stage or state came on, the administration of the most powerful antiseptics was unavailing. More than half of those thus diseased therefore perished.

"On dissection, the stomach and all the intestinal canal appeared inflamed, and generally covered with large livid blotches; and in the valvulæ conniventes was a considerable quantity of a yellow gelatinous matter. Large quantities of the same matter were found among the muscles of the abdomen, and between them and the peritonæum. The brain and all the other viscera were sound.

"The principal, and indeed only beneficial remedies, were bark and wine, exhibited before the commencement of the second state."

The ingenious author next traces the history given of a similar disease in the island of Barbadoes, first from the account given by the Rev. Mr. Hughes, and next from the information he (Dr. Chisholm) received, when making a tour of the island, from his friend and host Mr. Cummings. All the accounts agree in the contagious property of the disease, and its communication to the human, by eating or touching the flesh of the dead cattle, and, in one instance, by taking an over quantity of the milk from a diseased cow.

Having described, in the above manner, the disease between the tropics, Dr. C. proceeds to trace the similarity between it and the murrain of European cattle. In this he shows equal industry, and having every where written documents to refer to, is much more minute. In the northern disease he discovers two very striking symptoms, unnoticed between the tropics, viz. a pustular eruption on the fifth day as characteristic of the disease, and a peculiar swelling in the part, called by Sauvaage the *omasum*, and by Ramazzini *antica parte pectoris*. The part alluded to seems to have been the coat of fat under the integuments covering the parieties of the superior part of the abdomen, or the inferior part of the thorax. By each the tumor is called *anthrax*, but by the latter, on account of its situation, it is also denominated *anticardia*.

Ramazzini, who is much the most minute in his description of the symptoms, is not less so in his account of the appearances after death. He speaks of hydatids in different parts of the body, and also of bladders filled with air, and others filled with serum.

Had this faithful writer been more in the habit of examining the entrails of cattle, he would have found many of these appearances in animals, slaughtered apparently in health. They are therefore no further to be taken into the account, than as they may imply a probability that animals, in reduced health from any cause, may perhaps be more liable to encysted tumors.

But the most important difference in the two is, that if we can trust trust to the above accounts, the tropical disease was communicated to the human, not only by the application of sanious matter, but by eating the flesh of the dead cattle, and, in one instance, by the milk of a diseased cow. Whereas, by the testimony of Goelick and Haller, as cited by our author; and by the silence of Vicq d'Azyr and Dr. Layard, there is every reason to suppose that the murrain did not affect the human race. The opinions of Sauvaage, and the imperfect accounts of Lancisi, though tending to a different conclusion, are shown to be very unsatisfactory.

In remarking the difference in the symptoms between the European and intertropical cattle, the author conceives much, if not the whole, may be imputed to the difference of climate: every disease being for the most part more rapid in proportion as we approach the sun. Hence the cattle in the islands died before the full evolution of all the phænomena of the disease. We conceive the different accounts of the contagion reaching the human race may be explained with still more ease. The accounts of those who perished from eating the flesh, is extremely confused. Perhaps it stands on no better authority than Lancisi's remark, that those men in Rome who ate of the infected flesh were infected with diarrhœa and fever, which is contrary to the testimony of most contemporary writers. That others might be infected by the effluvia, or contact with diseased parts, is not only probable, but an occurrence that might happen under any other local disease, or by contact with flesh in a putrid state from the common process of corruption.

Our industrious author next enters into a long disquisition on the subject of diseases communicated from one race to another, or rather of their existing at the same time in different species of animals. In the account above-mentioned by himself, there was a coincidence of angina maligna and the pestis bovina at Grenada; yet he candidly acknowledges not only the difficulty of tracing on many occasions any immediate means of infection, but even of ascertaining whether the cynanche maligna in the human subject preceded or followed the epizootic malady. That pestilential diseases have at the same time affected various classes of animals and man cannot be questioned; but under such circumstances, there is every reason to suppose that the whole originated in some constitution of the atmosphere, even admitting that the disease became afterwards infectious. The author concludes his paper with the following paragraph.

"Having lengthened this paper much beyond the limits I originally proposed, I shall intrude no longer at present; but as the inquiry involves in it a very curious and important question relative to the influence of the effluvia from dead animal bodies, passing through the natural process of putrefaction in the open air, on living animal bodies, I shall take some future occasion to communicate to you such remarks as have occurred to me on the subject."

We shall feel our obligations much increased, if Dr. Chisholm will in his future paper, accurately distinguish between diseases communicated by contagion from one race of animals to another, and those which may be imputed to a common cause in the atmosphere, and if in the former he would distinguish such as were excited only by the contact of diseased matter from those which may be imputed to the contact with mere putrid matter. We are aware of the difficulties attending these inquiries, and on that account we impose them with the less reluctance on a writer of some leisure, and of extensive means of information, both from reading and actual observation.

( To be continued. )

